# Burden of influenza during the first year of life

**DOI:** 10.1111/irv.12820

**Published:** 2020-10-18

**Authors:** Janna‐Maija Mattila, Emilia Thomas, Pasi Lehtinen, Tytti Vuorinen, Matti Waris, Terho Heikkinen

**Affiliations:** ^1^ Department of Pediatrics University of Turku and Turku University Hospital Turku Finland; ^2^ Department of Clinical Microbiology Turku University Hospital, and Institute of Biomedicine University of Turku Turku Finland

**Keywords:** child, infant, influenza, otitis media

## Abstract

**Background:**

Every year, influenza viruses infect millions of children and cause an enormous burden of disease. Young children are at the highest risk for influenza‐attributable hospitalizations. Nevertheless, most young children are treated as outpatients, and limited data are available on the burden of influenza in these children.

**Methods:**

We carried out a prospective cohort study and followed 431 infants born in June‐August 2017 for 10 months from September 1, 2017, to June 30, 2018. The parents filled out daily symptom diaries and were instructed to bring their child for clinical examination at our study clinic each time the child had fever or any signs or symptoms of respiratory tract infection. During each visit, we obtained nasopharyngeal swab specimens for determination of the viral etiology of the illness.

**Results:**

A total of 55 episodes of laboratory‐confirmed influenza were diagnosed among the 408 actively participating children, which corresponds to an annual incidence rate of 135/1000 children (95% Cl, 102‐175). Excluding five children with double viral infection, acute otitis media developed as a complication of influenza in 23 (46%) children. One (2%) child with influenza was hospitalized because of febrile convulsion. The effectiveness of influenza vaccination was 48% (95% CI, −29%‐80%).

**Conclusions:**

The burden of influenza during the first year of life is heavy in the outpatient setting where most infants with influenza are managed. Effective strategies for the prevention of influenza particularly in infants under 6 months of age are needed to diminish the burden of disease in this age group.

## 1 INTRODUCTION

Every year, seasonal influenza viruses are estimated to cause globally >100 million episodes of illness and 870 000 hospitalizations for acute lower respiratory infection among children under 5 years of age.^1^ Children under 1 year of age are at the highest risk for influenza‐attributable hospitalizations, and the youngest infants are frequently admitted due to sepsis‐like symptoms.^2^, ^3^, ^4^, ^5^, ^6^, ^7^ Influenza‐associated childhood mortality rates are also highest among the youngest children.^8^, ^9^ Furthermore, children have a central role in the transmission of influenza in the community.^10^, ^11^

Prevention of influenza in young infants is difficult. Influenza vaccines are licensed for use only in children older than 6 months of age, and the response to influenza vaccination in young infants is generally weaker than in older children.^12^ Maternal influenza vaccination during pregnancy reduces the risk of influenza in infants, but the duration of protection afforded by maternal antibodies is limited.^13^, ^14^ Because most infants probably contract influenza from their family members, influenza vaccination of the other family members might provide some protection for infants too young to be vaccinated themselves.^15^ In many cases, however, the only way of reducing the burden of influenza in infants is by ameliorating the illness by the use of oseltamivir treatment.^16^, ^17^, ^18^

Although the high rates of influenza‐associated hospitalization among young infants have been described in several studies,^2^, ^3^, ^4^, ^5^, ^6^ most young infants are seen and treated as outpatients. Only limited data exist on the clinical features and overall burden of influenza among outpatient children during their first year of life.^19^, ^20^ The aim of this study was to assess the incidence, clinical presentation, and complications of influenza virus infections in a prospectively followed cohort of newborn infants during their first influenza season.

## 2 METHODS

### 2.1 Study design

This prospective cohort study was performed at a primary care study clinic in Turku, Finland, from September 1, 2017, to June 30, 2018. Before the study period, 431 children born in June‐August 2017 at Turku University Hospital were enrolled in a follow‐up cohort. The parents of newborn children received written information about the study at the maternal ward of the hospital soon after the child was born, and those who wanted their child to participate signed an informed consent form. Of all children born during the enrollment period, approximately half were enrolled in this study. The study protocol was approved by the Ethics Committee of the Hospital District of Southwest Finland (approval number 47/1801/2017).

### 2.2 Study conduct

The study clinic was open every day during the 10‐month study period, including weekends and holidays. The parents were instructed to bring their child for clinical examination at the study clinic as soon as possible after the onset of fever or any signs of respiratory infection. During each visit, a study physician examined the child, filled out a structured medical record, and obtained nasopharyngeal swab specimens for determination of the viral etiology of the illness. The structured medical record contained detailed questions about the presence and duration of the child´s preceding signs and symptoms and detailed findings at the clinical examination. Oseltamivir treatment was given to all children who were diagnosed with a laboratory‐confirmed influenza within 48 hours of illness onset. The dosage of oseltamivir was 3 mg/kg twice daily for 5 days.

The children were routinely re‐examined on days 5‐7 after the onset of illness and additionally whenever the parents deemed it necessary, especially if they suspected the worsening of the disease or the development of a complication such as acute otitis media (AOM). All visits were free of charge to the families, and there was no limit for the number of visits during the study.

Background information regarding the family, pregnancy, and delivery was obtained from the parents of all children. The parents were provided with symptom diaries (one for September‐January and another for February‐June) that they were asked to complete daily throughout the 10‐month follow‐up period. Children were considered active participants if they visited the study clinic at least once or if the parents returned at least one of the two symptom diaries, and if the parents did not inform the study personnel that their child had been treated for a respiratory illness somewhere else than at the study clinic. The baseline characteristics of the 408 active participants are shown in Table 1.

**TABLE 1:** Baseline characteristics of the 408 actively participating children

Characteristic	No. of children	%
Sex		
Male	208	51.0
Female	200	49.0
Age at start of follow‐up (mo)		
<1	133	32.6
1‐<2	163	40.0
2‐<3	112	27.5
Birth weight (g)		
<2500	23	5.6
2500‐3999	313	76.7
≥4000	72	17.6
Method of delivery		
Vaginal	349	85.5
Cesarean section	59	14.5
Gestational age (wk)		
<37	27	6.6
37‐41	367	90.0
≥42	14	3.4
No. of siblings		
0	178	43.6
1	142	34.8
≥2	88	21.6
Parental smoking	77	18.9
Influenza vaccination	54	14.2^a^

^a^ Data on vaccination were available for 379 children.

### 2.3 Definitions

The overall duration of illness consisted of all consecutive days on which the child had fever, rhinitis, or cough. The diagnosis of AOM was based on the presence of middle‐ear effusion and signs of inflammation of the tympanic membrane as detected by pneumatic otoscopy, together with at least one sign of an acute infection. Data on influenza vaccination of the family members were collected after the influenza season. Children in the follow‐up cohort who had received two doses of seasonal influenza vaccine were classified as vaccinated. The trivalent inactivated influenza vaccine for use in the 2017‐2018 influenza season contained the following strains: A/Michigan/45/2015; A/Hong Kong/4801/2014; and B/Brisbane/60/2008.

### 2.4 Virologic methods

Two nasopharyngeal flocked swab specimens (Ultra minitip, Copan Italia S.p.a, Italy) were collected for viral analyses from each child at the initial visit for each respiratory infection. One of the specimens was analyzed onsite at the study clinic by rapid antigen detection (mariPOC^®^ respi test, ArcDia International Ltd, Finland), and the other specimen was analyzed by reverse‐transcription polymerase chain reaction (RT‐PCR) at the Department of Clinical Microbiology, Turku University Hospital (Allplex^TM^ Respiratory Panels 1‐3, Seegene Inc, South Korea). The order of sampling for the two tests was not standardized. The specimens for RT‐PCR assays were refrigerated until analyzed in the virologic laboratory the next working day. A child was considered to have influenza if any of the specimens tested positive for influenza A or B viruses by either of these methods and if no other virus dominated in the sample as determined by the cycle threshold (Ct) values for different viruses in the multiplex RT‐PCR analyses. The Ct value is defined as the calculated cycle number at which the PCR product crosses a threshold of detection, and it provides a semiquantitative measure of viral load.

### 2.5 Statistical analyses

Comparison of proportions between two groups was performed by the chi‐square test or Fisher´s exact test, and medians were compared by the Mann‐Whitney *U* test. All tests were 2‐sided, and *P* values <.05 were considered to indicate statistical significance. The incidence rate of influenza was calculated by dividing the number of influenza episodes by the follow‐up time. Considering the well‐known annual epidemiology of influenza and the need to provide a conservative estimate, the follow‐up time for each child was determined as 1 year, although the actual follow‐up time was 10 months. All statistical analyses were performed with StatsDirect software (version 3.2.7; StatsDirect Ltd, Cambridge, UK).

## 3 RESULTS

### 3.1 Incidence of influenza

**FIGURE 1:**
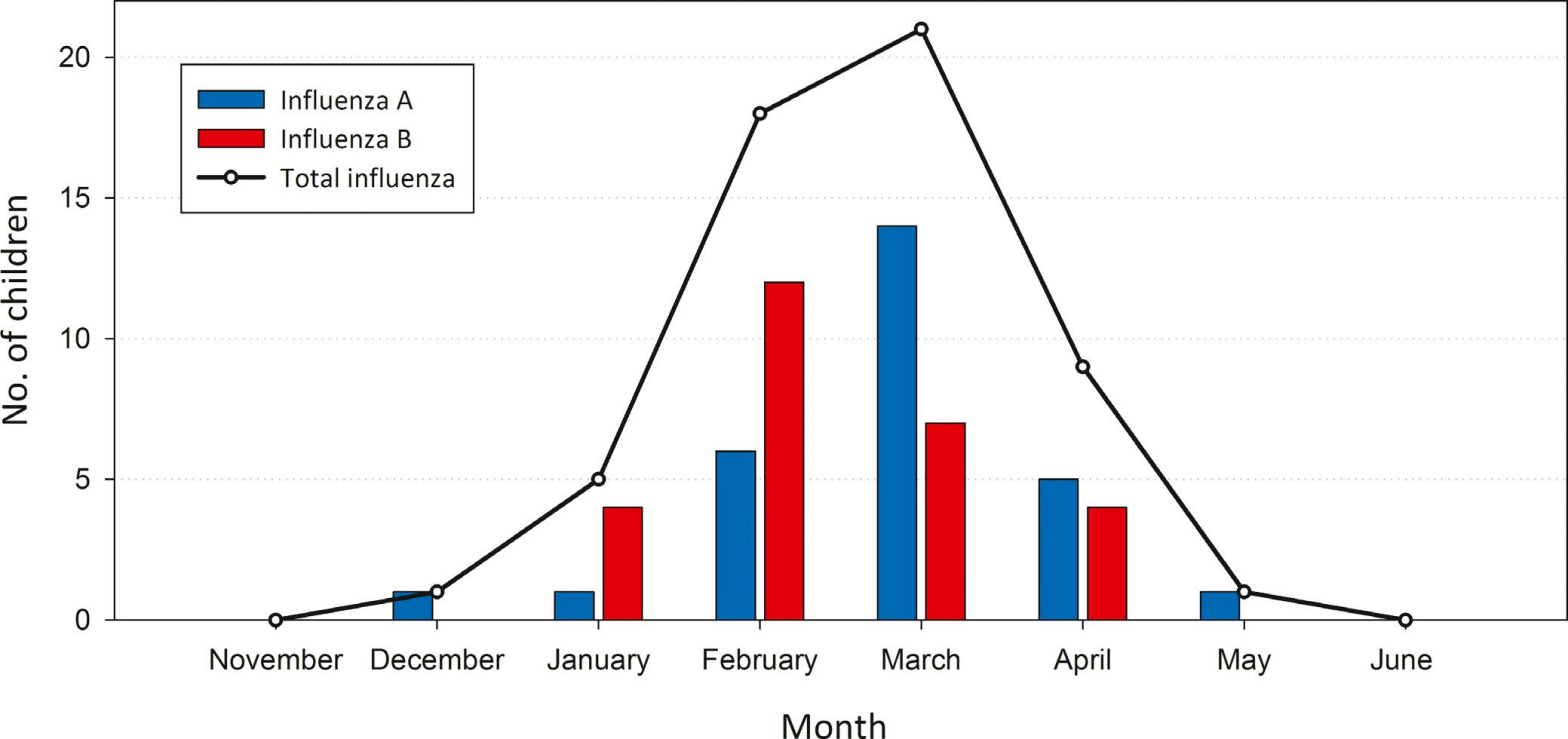
Monthly detections of influenza A and B virus infections in the study cohort during the influenza season of 2017‐2018

A total of 55 episodes of laboratory‐confirmed influenza were diagnosed among the 408 actively participating children, which corresponds to an annual incidence rate of 135/1000 children (95% Cl, 102‐175). Of these 55 episodes, 28 (50.9%) were caused by influenza A viruses and 27 (49.1%) by influenza B viruses; all cases were positive by RT‐PCR. The monthly detections of influenza A and B viruses in the follow‐up cohort are shown in Figure 1. During the influenza season of 2017‐2018 in Finland, the dominant virus types were A/H3N2 (41% of all) and B/Yamagata (54% of all) viruses.

One child had two separate influenza episodes, one caused by influenza A and another by influenza B viruses. Of the 54 children (13.2% of all) with at least one episode of influenza, 26 (48.1%) were boys and 28 (51.9%) girls. Thirty‐three (61.1%) children had at least one sibling, and 4 (7.4%) had received two doses of seasonal influenza vaccine. The mean age of the children at the diagnosis of influenza was 7.6 months (range, 4.3‐11.0).

### 3.2 Clinical presentation at the initial visit

Five episodes of influenza were excluded from all analyses of clinical findings because of confirmed double viral infections in which influenza A or B virus was not the predominant virus (rhinovirus, 3 cases; respiratory syncytial virus, 2 cases). Among the remaining 50 influenza episodes, influenza A virus was detected in 26 (52.0%) and influenza B virus in 24 (48.0%) cases.

The signs and symptoms and clinical findings at the initial visit in children with influenza are presented in Table 2. Overall, 48 (96.0%) of 50 children had some fever, and 23 (46.0%) had fever ≥39.0°C; the mean maximum fever at or before the initial visit for influenza was 38.8°C. No child had a sepsis‐like clinical presentation. Rhinitis was present in 41 (82.0%) children, and 37 (74.0%) children had cough. Gastrointestinal symptoms were present in 11 (22.0%) children. None of the differences between influenza A and B infections were statistically significant.

**TABLE 2:** Signs and symptoms and clinical diagnoses at the initial visit in 50 children with influenza^a^

Variable	Influenza A (n = 26)	Influenza B (n = 24)	Any influenza (n = 50)
Fever ≥ 37.5°C	25 (96)	23 (92)	48 (96)
Fever ≥ 38.0°C	23 (88)	20 (83)	43 (86)
Fever ≥ 39.0°C	13 (50)	10 (42)	23 (46)
Rhinitis	19 (73)	22 (92)	41 (82)
Cough	22 (85)	15 (63)	37 (74)
Vomiting	4 (15)	2 (8)	6 (12)
Diarrhea	2 (8)	4 (17)	6 (12)
Any gastrointestinal symptom	6 (23)	5 (21)	11 (22)
Conjunctivitis	3 (12)	2 (8)	5 (10)
Expiratory wheezing	0 (0)	2 (8)	2 (4)
Acute otitis media	6 (23)	6 (25)	12 (24)

^a^ Data are n (column %).

### 3.3 Duration of symptoms

The median duration of preceding symptoms before the initial visit was 17.6 hours (interquartile range [IQR], 9.5‐43.0). The duration of preceding symptoms was 13.5 hours (IQR, 7.5‐23.8) in children with influenza A and 26.8 hours (IQR, 11.3‐76.4) in children with influenza B (*P* = .035). The overall median duration of illness in children with influenza was 8.0 days (IQR, 6.0‐11.3), and the median duration of fever was 3.0 days (IQR, 1.8‐4.0); no differences were observed between influenza A and B illnesses.

### 3.4 Complications and management

The most frequent complication of influenza was AOM which was diagnosed in 23 (46.0%) of 50 children; 11 (47.8%) of all 23 cases of AOM were diagnosed during the follow‐up visits after the initial clinical examination. None of the children had pneumonia. A total of 21 (42.0%) children were treated with antibiotics; all treatments were for AOM. One (2.0%) child was hospitalized because of febrile convulsion.

Overall, 40 (80.0%) of 50 children with influenza came to the initial visit within 48 hours of the onset of symptoms, and influenza was virologically confirmed during the first 48 hours of symptoms in 37 (74.0%) children; all these children received oseltamivir treatment for 5 days. One child with influenza was cared for outside home and was absent from day care because of influenza.

### 3.5 Effectiveness of influenza vaccination

Data on seasonal influenza vaccination in the family were available for 379 children. Of the 54 children who had been vaccinated against influenza, 4 (7.4%) were diagnosed with influenza. Among the 325 unvaccinated children, influenza was diagnosed in 46 (14.2%) cases, corresponding to a vaccine effectiveness of 48% (95% Cl, −29%‐80%; *P* = .17). The type‐specific effectiveness of vaccination was 52% (95% CI, −72%‐87%; *P* = .29) against influenza A and 45% (95% CI, −97%‐86%; *P* = .39) against influenza B. Of the 325 unvaccinated children, 123 (37.8%) lived in households in which all other family members had received influenza vaccination for the season. Seventeen (13.8%) of these 123 children were diagnosed with influenza, compared with 29 of 202 (14.4%) children living in households in which at least one of the other family members had not been vaccinated (vaccine effectiveness 4%; 95% CI, −66%‐45%; *P* = .89). The effectiveness of vaccination against influenza‐associated AOM was 5% (95% CI, −182%‐70%; *P* = .93).

## 4 DISCUSSION

This prospective cohort study provides detailed data about the clinical features and overall burden of influenza in a large and representative group of young infants followed carefully throughout their first influenza season. Although virtually all infants in the cohort were taken care of at home, 13% of them contracted a laboratory‐confirmed influenza. In almost half of children with influenza, the course of illness was complicated by the development of AOM that was mostly treated with antibiotics. Our study also confirms that the greatest part of the total burden of influenza even among the youngest children occurs in the outpatient setting. Although none of the infants had had prior exposure to influenza viruses and only a minor proportion of them were vaccinated, only one child was hospitalized with influenza.

Our observed incidence rate of influenza during the first year of life is comparable with previous results among older children that have demonstrated annual influenza attack rates of 10%‐20% in children.^21^, ^22^ In one recent follow‐up study, the incidence of laboratory‐confirmed influenza among young children during their first influenza season was only 5%.^20^ One potential explanation for the difference is in the study design; in that previous study, substantial numbers of nasal swab samples were obtained at home by the parents who mailed the specimens to the laboratory, and eventually, a specimen for viral diagnosis was available for approximately half of all respiratory infections in the cohort. However, it is also well‐known that influenza epidemics vary in intensity and severity, and the attack rates could be lower than average during any milder influenza seasons.

In this study, AOM was diagnosed as a complication of influenza in 46% of young children, which is in agreement with previous studies and demonstrates that influenza viruses belong to the most important viruses predisposing children to AOM.^21^, ^23^, ^24^ It is worth noticing that about half of all cases of AOM developed after the first visit to the study clinic. This is in line with studies showing that the incidence of AOM peaks on days 3‐4 after the onset of symptoms of a viral respiratory infection,^24^, ^25^, ^26^ and it emphasizes the importance of clinical re‐examinations to capture all cases of AOM.

In previous studies, fever has been the strongest sign associated with influenza^20^, ^27^, ^28^, ^29^ and that applied also to the infants in our study. However, as observed also previously, rhinitis and cough were frequently present already in the early phase of the illness.^27^, ^29^ This corroborates earlier conclusions that especially in young children influenza virus infections are clinically indistinguishable from other respiratory viral infections, and they cannot be reliably identified without the use of specific diagnostic tests.^30^, ^31^

The duration of symptoms before the initial visit to the study clinic was shorter in children with influenza A than in those with influenza B. It is therefore possible that the early symptoms of influenza A infection are more prominent in infants than symptoms caused by influenza B viruses. Although this finding suggests a need for further studies, it is also possible that the observed difference was due to chance only, because ample recent data indicate that there are no differences in the clinical presentation of influenza A and B virus infections in children.^32^, ^33^, ^34^, ^35^ Furthermore, no other differences between influenza A and B were observed in this study.

Although the point estimate of the effectiveness of influenza vaccination of infants in our study was 48%, it was not statistically significant. This is in line with the nationwide analysis conducted by the Finnish Institute for Health and Welfare, concluding that no effectiveness could be shown for the trivalent inactivated influenza vaccine used during the season of our study.^36^, ^37^ Because vaccine effectiveness was low not only in children but also in adults, it could well explain why vaccination of all other family members in our study did not reduce the incidence of influenza in the infants. In the absence of an influenza vaccine that could be administered to infants under 6 months of age, many health authorities emphasize the importance of vaccinating all household members and other close contacts to protect infants from influenza.^15^ It is important to notice that our results do not demonstrate that such a “cocooning” strategy would not work in principle. It is just an inconvenient fact that during seasons when there is a substantial mismatch between the vaccine and circulating strains of influenza, no major benefit, either direct or indirect, can be expected from vaccination against influenza.

The strengths of our study include close follow‐up of a large cohort of infants who were enrolled without any exclusion criteria, easy and free access to the study clinic that was open every day during the 10‐month study period, careful clinical examination and routine re‐examinations during each illness, collection of nasopharyngeal swabs during each illness irrespective of the severity of symptoms, and daily recording of the infants´ symptoms in the diaries by the parents. The main limitation of our study is that it covered only a single influenza season; conducting studies with similar setup during several consecutive seasons would increase the accuracy of the estimates. Furthermore, the final numbers of children with influenza were relatively small, which resulted in reduced power to show statistically significant differences especially between influenza A and B viruses.

In conclusion, our study provides new and detailed information about influenza in young infants during their first influenza season. Besides the relatively high rates of influenza‐associated hospitalization among the youngest infants, the burden of illness is heavy also in the outpatient setting where most infants with influenza are managed. Because influenza vaccines are licensed only for children older than 6 months and the duration of protection afforded by maternal antibodies is limited, effective strategies for the prevention of influenza particularly in infants under 6 months of age are needed to diminish the burden of disease in this age group.

## CONFLICTS OF INTEREST

TH has received consulting fees from AstraZeneca, Sanofi Pasteur, and Seqirus. The other authors have no conflicts to report.

## AUTHOR CONTRIBUTION

**Janna‐Maija Mattila:** Conceptualization (supporting); Data curation (equal); Formal analysis (equal); Investigation (equal); Methodology (equal); Writing‐original draft (lead); Writing‐review & editing (supporting). **Emilia Thomas:** Investigation (equal); Writing‐review & editing (supporting). **Pasi Lehtinen:** Investigation (equal); Writing‐review & editing (supporting). **Tytti Vuorinen:** Methodology (equal); Writing‐review & editing (supporting). **Matti Waris:** Methodology (equal); Writing‐review & editing (supporting). **Terho Heikkinen:** Conceptualization (lead); Data curation (equal); Formal analysis (equal); Funding acquisition (lead); Methodology (equal); Project administration (lead); Supervision (lead); Writing‐review & editing (lead).

### PEER REVIEW

The peer review history for this article is available at https://publons.com/publon/10.1111/irv.12820.

## Data Availability

The data that support the findings of this study are available on request from the corresponding author. The data are not publicly available due to privacy or ethical restrictions.
